# Performances of clinical characteristics and radiological findings in identifying COVID-19 from suspected cases

**DOI:** 10.1186/s12880-022-00780-y

**Published:** 2022-03-26

**Authors:** Xuanxuan Li, Yajing Zhao, Yiping Lu, Yingyan Zheng, Nan Mei, Qiuyue Han, Zhuoying Ruan, Anling Xiao, Xiaohui Qiu, Dongdong Wang, Bo Yin

**Affiliations:** 1 Department of Radiology, Huashan Hospital, Fudan University, 12, Middle Wulumuqi Rd., Jing’an District, Shanghai, 200040 China; 2 Department of Radiology, Fu Yang No. 2 People’s Hospital, 450 Linquan Road, Fuyang, Anhui Province China; 3 Department of Radiology, Bozhou People’s Hospital, 616, Duzhong Road, Bozhou, Anhui Province China

**Keywords:** COVID-19, Differential diagnosis, X-ray computed tomography, Logistic models, Nomograms

## Abstract

**Background:**

To identify effective factors and establish a model to distinguish COVID-19 patients from suspected cases.

**Methods:**

The clinical characteristics, laboratory results and initial chest CT findings of suspected COVID-19 patients in 3 institutions were retrospectively reviewed. Univariate and multivariate logistic regression were performed to identify significant features. A nomogram was constructed, with calibration validated internally and externally.

**Results:**

239 patients from 2 institutions were enrolled in the primary cohort including 157 COVID-19 and 82 non-COVID-19 patients. 11 features were selected by LASSO selection, and 8 features were found significant using multivariate logistic regression analysis. We found that the COVID-19 group are more likely to have fever (OR 4.22), contact history (OR 284.73), lower WBC count (OR 0.63), left lower lobe involvement (OR 9.42), multifocal lesions (OR 8.98), pleural thickening (OR 5.59), peripheral distribution (OR 0.09), and less mediastinal lymphadenopathy (OR 0.037). The nomogram developed accordingly for clinical practice showed satisfactory internal and external validation.

**Conclusions:**

In conclusion, fever, contact history, decreased WBC count, left lower lobe involvement, pleural thickening, multifocal lesions, peripheral distribution, and absence of mediastinal lymphadenopathy are able to distinguish COVID-19 patients from other suspected patients. The corresponding nomogram is a useful tool in clinical practice.

## Introduction

In December 2019, a few pneumonia cases of unknown etiology were reported in Wuhan, Hubei Province, China [1]. The disease, now named coronavirus disease 2019 (COVID-19) then spread at a striking speed worldwide. The causative organism was identified as a novel coronavirus named severe acute respiratory syndrome coronavirus 2 (SARS-CoV-2) due to the phylogenetic similarity to SARS-CoV [2]. As of October 15th, 2022, there were a total of 238,940,176 cumulative cases and 4,882,066 cumulative deaths worldwide. COVID-19 was declared as a public health emergency of international concern (PHEIC) by the World Health Organization (WHO) as early as January 30th, 2020 [3, 4].

The confirmation of COVID-19 relies on the positive result of the nucleic acid amplification test (NAAT) of the upper respiratory tract specimens using the ﻿real-time reverse transcriptase–polymerase chain reaction (RT-PCR) tests [5]. However, the limitations of RT-PCR tests include: 1) The severity and progression of the disease cannot be quantitatively judged. 2) They have long turnaround times, especially in less developed regions. 3) They require certified laboratories, expensive equipments and trained technicians [6, 7].

On the contrary, chest CT scan is relatively easy to perform with fast diagnosis and the sensitivity reached as high as 97% for COVID-19 according a study of 1014 patients in Wuhan [8]. Chest CT abnormalities have also been identified in patients even prior to the development of symptoms or the detection of viral RNA [9, 10]. Thus it has a great value in early identification of COVID-19 [8, 11, 12]. Chest CT imaging is also a useful tool in monitoring COVID-19 progression and therapeutic effect in clinical settings [13]. The Diagnosis and Treatment Program of COVID-19 (trail version 8) [14, 15] formulated by the National Health Commission of China has summarized the typical CT manifestations of COVID-19 as follows and incorporated them in the diagnosis criteria: multiple small patchy shadows and interstitial changes are seen, mainly in periphery lungs. This may progress into bilateral multiple ground glass opacities (GGOs) and infiltrations. In severe cases, consolidation may occur, but pleural effusion is rare. In multiple system inflammatory syndrome (MIS-C), patients with cardiac insufficiency can show enlarged heart silhouette and pulmonary edema.

Patients with above-said CT manifestations are suspected as COVID-19 infectors therefore need further examinations. Before the RT-PCR result is available, the patient needs isolation, but the quarantine of the patients may lead to a waste of medical resources and a possible delay of essential treatment. Hence, effective and convenient methods to better distinguish COVID-19 patients are needed.

The aim of our study is to identify the useful clinical, laboratory and radiographic features that are able to distinguish COVID-19 patients from other suspected cases and generate a nomogram as a useful tool for clinical practice.

## Materials and methods

**Fig. 1:**
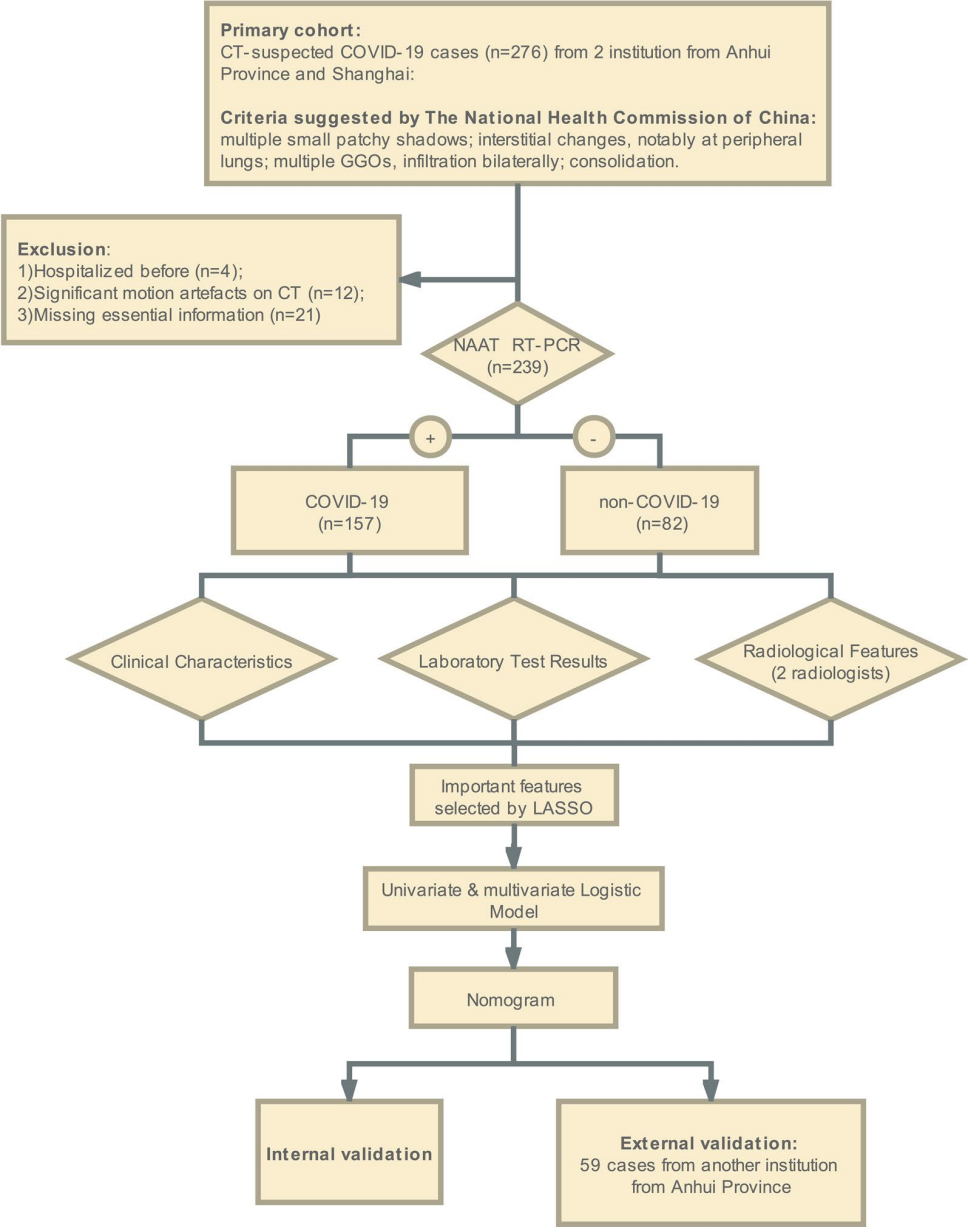
Workflow of the whole study

The schematic workflow is depicted in Figure 1.

### Patient cohort

Data were de-identified to guarantee the patients’ confidentiality. From January 21th to March 5th, 2020, patients admitted to a hospital in Anhui province, China and our institution in Shanghai, China who met the following requirements were enrolled as the primary cohort in our study: (1) Patients with chest CT manifestations suggested by the Diagnosis and Treatment Program of COVID-19 (trail version 8) [15] that had a suspicion of COVID-19. (2) Patients that took laboratory examination at admission. (3) Patients diagnosed of COVID-19 with positive RT-PCR for SARS-CoV-2, or patients excluded from the diagnosis of COVID-19 with 2 consecutively negative RT-PCR test results separated by at least 1 day. Exclusion criteria included: (1) Patients who were hospitalized before (n = 4). (2) Significant motion artefacts in CT images (n = 12). (3) Patients lacking essential data (n = 21). The epidemiological history, the symptoms, the laboratory test results and the imaging features of their first CT scan after onset were recorded.

From February 6th to March 13th, 2020, an independent cohort of CT-suspected patients from another institution in Anhui Province was retrospectively studied, using the same inclusion and exclusion criteria. These patients formed the validation cohort.

The laboratory tests were carried out in the outpatient department or in the wards on admission, mostly on the same day when CT scan was done. Collected laboratory indices included the white blood cell (WBC) count, lymphocyte count, lactate dehydrogenase (LDH), C reactive protein (CRP), procalcitonin (PCT), alanine aminotransferase (ALT), and aspartate aminotransferase (AST).

### CT protocol

105 patients from Huashan Hospital Affiliated to Fudan University were imaged with 1.5 mm-thickness with a 256-slice spiral CT scanner (Philips). 134 patients from Fuyang No.2 People`s Hospital were imaged with 1 mm-thickness with a 64-section CT scanner (Aquilion 64, Toshiba Medical Systems). 59 patients from Bozhou People`s Hospital in the validation cohort were imaged with 5 mm-thickness with a 64-section CT scanner (Siemens Somantom Sensation).

### CT manifestation analysis

All imaging data were analyzed with consensus by two experienced radiologists (Y.L. and D.W., general radiologists with 12 and 7 years of experience in CT interpretation). 23 features from 18 aspects were collected as listed below: (a) The involved pulmonary lobes including five features: right upper, right middle, right lower, left upper, left lower lobes; (b) Distribution of lesions including two features: anterior and posterior part of lungs; (c) The location of lesions that is set as dummy variables: peripheral (the outer one-third of the lung), central (the inner two-thirds of the lung) or both; (d) The extent of the lesions that is set as dummy variables: unifocal (only one lesion can be observed), multifocal (multiple lesions separated from each other by uninvolved lung tissue) and diffuse (dispersed over a large area). (e) An extent score was semi-quantitatively calculated. Both lungs were divided into upper (above tracheal carina), lower (below inferior pulmonary vein) and middle (in between) zones, and involved percentage in each zone was scored: 0, 0%; 1, < 25%;2, 25%—49%; 3, 50%—74%; 4, > 75%, and they added up to the extent score (range 0–24). (f) The existence of opacification set as dummy variables included GGO, mixed (mainly GGO), mixed (mainly consolidation) and consolidation; (g) The shape of the lesions, including nodular (characterized by a rounded or irregular opacity, well or poorly defined, measuring up to 3 cm in diameter), linear (fine linear opacity), patchy (isolated focal lesions with no nodular/linear shape in the segment) and large patchy (large fused lesions involving multiple segments); (h) The halo sign; (i) The reversed halo sign; (j) Reticulated changes; (k) The existence of vascular enlargement; (l) The existence of air bronchogram; (m) Bronchiectasis; (n) Pleural thickening (> 3 mm); (o) Pleural traction; (p) Pleural effusion; (q) Mediastinal lymphadenopathy (the short axial diameter > 1 cm); (r) Liver spleen ratio (LS ratio) was calculated as CT_liver_/CT_spleen_ to indicate the relative density. Five 1cm^2^ regions of interests (ROI) were drawn in the liver and spleen parenchymal to obtain the mean CT values of liver and spleen. The description of the radiological features of the lungs followed the definition compiled by the Fleischner Society [16].

### Feature selection

The clinical [8], laboratory [7] and CT features [23] were analyzed altogether, but with the limited sample size, a total of 38 features would lead to overfitting in multivariate analysis. Thus, the least absolute shrinkage and selection operator (LASSO) method was adopted to select the most relevant features. This method is able to shrink the coefficients and diminish some to zero, thus can be used for feature reduction and selection. The R software and the “glmnet” package (version 3.6.0; R foundation for Statistical computing) were used.

### Statistical analysis

All statistical analyses were executed with R software. The Shapiro–Wilk test was used to evaluate the distribution type and Bartlett`s test was used to evaluate the homogeneity of variance. Normally distributed data were displayed as mean ± standard deviation. Non-normally distributed data and ordinal data were displayed as median (inter-quartile range). Categorical variables were summarized as counts and percentages. Both univariate and multivariate logistic regression were analyzed to demonstrate the correlation of the features with COVID-19 diagnosis. The regression coefficient (β) was calculated using the odds ratio (OR). The model was estimated as follows:

$$\upbeta =\mathrm{log}\left(\mathrm{OR}\right)$$

$$\mathrm{logit P}={\upbeta }_{1}{\upchi }_{1}+{\upbeta }_{2}{\upchi }_{2}+\dots +{\upbeta }_{i}{\upchi }_{i}$$

A nomogram was established. The calibration ability was internally assessed with the bootstraping method and the Hosmer–Lemeshow test (HL test) was performed to test the goodness of fit.

For the external validation of the nomogram, the prediction value of each case was calculated according to the nomogram and compared with the observed diagnosis. The accuracy was validated by correctly predicted case proportion and the HL goodness-of-fit test. A *P*-value of < 0.05 was defined as statistical significance.

### IRB approval

This multi-center retrospective study was approved by the institutional review board (IRB) and the requirement of written informed consent was waived.

## Results

### Clinical information

The clinical information, laboratory tests, and chest CT imaging findings were compared between the primary cohort and validation cohort (Tabled 1 and 2). In the primary cohort, 239 patients (134 males and 105 females) were included in this study with an average age of 46.31 ± 15.90 years old. 28.87% of the patients had a direct contact with confirmed COVID-19 patients before the onset or had travelled/lived in the Hubei Province. 17.57% of the patients had indirect contact. Most common symptoms the patients presented were fever (70.29%), cough (44.35%), and chest distress (11.30%). Some patients had digestive symptoms such as diarrhea (2.09%) and anorexia (2.09%) (Table 1). The median interval between the onset and the date of CT scan was 8 (range 1–22) days. 157 patients were confirmed as COVID-19 by RT-PCR and were allocated to the COVID-19 group. They were put in quarantine and treated with the antiviral therapy based on the evolving recommendations [17]. The other 82 patients had negative RT-PCR results. They were eventually diagnosed as other conditions such as viral pneumonia (influenza type A virus, respiratory syncytial virus), bacterial infection (*Staphylococcus aureus, Streptococcus pneumoniae*), fungal infection (*pneumocystis jiroveci* pneumonia), *mycoplasma pneumoniae* pneumonia, and other respiratory conditions (acute eosinophilic pneumonia, Goodpasture syndrome etc.). Clinical information of two groups were compared using univariate analysis (Table 3). COVID-19 patients were found to be younger (*P* = 0.037), more likely to have fever (*P* = 0.001) or cough (*P* < 0.001), and more likely to have contact history (*P* < 0.001).

**Table 1:** Clinical characteristics and laboratory tests of the primary cohort and validation cohort

Clinical characteristics	Primary cohort (n = 239)	Validation cohort (n = 59)	*P* value
Age, mean ± SD	46.30 ± 15.90	45.64 ± 16.57	0.614
Gender			
Male	134 (56.07%)	31 (52.54%)	0.733
Female	105 (43.93%)	28 (47.46%)	
Epidemiological history			
Direct contact	69 (28.87%)	19 (32.20%)	0.546
Indirect contact	42 (17.57%)	13 (22.03%)	
None contact	128 (53.55%)	27 (45.76%)	
Symptom			
Fever	168 (70.29%)	47 (79.66%)	0.202
Cough	106 (44.35%)	31 (52.54%)	0.097
Chest distress	27 (11.30%)	6 (10.17%)	0.988
Diarrhea	5 (2.09%)	5 (8.47%)	0.042*
Anorexia	5 (2.09%)	1 (1.69%)	1.000
Laboratory Test, median (inter-quartile range)			
WBC, median (range), × 10^9^/L	5.28 (4.30–10.44)	5.96 (3.91–6.00)	0.101
Lymphocyte count, median (range), × 10^9^/L	1.19 (0.90–1.63)	1.21 (0.85–1.44)	0.746
LDH, median (range), U/L	233.00 (193.00–271.40)	234 (199–290)	0.158
CRP, median (range), mg/L	14.80 (4.8–42.93)	25.90 (3.7–30.30)	0.038*
PCT, median (range), ng/mL	0.05 (0–0.19)	0.04 (0.02–0.06)	0.743
ALT, median (range), U/L	30.00 (20.00–51.50)	29.90 (17.30–37.70)	0.558
AST, median (range), U/L	28.00 (21.00–46.75)	28.00 (20.40–34.70)	0.450

WBC: White blood cell count; LDH: Lactate dehydrogenase; CRP: C-reactive protein; PCT: Procalcitonin; ALT: Alanine aminotransferase; AST: Aspartate aminotransferase

**Table 2:** Imaging manifestations on chest CT of the primary and validation cohort

Imaging manifestation	Primary cohort (n = 239)	Validation cohort (n = 59)	*P* value
Involved lobes			
Right Upper Lobe	144 (60.25%)	39 (66.1%)	0.498
Right Middle Lobe	129 (53.97%)	32 (54.24%)	1.000
Right Lower Lobe	179 (74.9%)	39 (66.1%)	0.230
Left Upper Lobe	143 (59.83%)	40 (67.8%)	0.329
Left Lower Lobe	176 (73.64%)	44 (74.58%)	1.000
Main distribution			
Anterior Part of Lungs	44 (18.41%)	18 (30.51%)	0.061
Posterior Part of Lungs	168 (70.29%)	40 (67.8%)	0.847
Location of lesions			
Peripheral	158 (66.11%)	33 (55.93%)	0.191
Central	16 (6.69%)	2 (3.39%)	0.516
Both	65 (27.2%)	24(40.68%)	0.482
Extent of lesions:			
Unifocal	58 (24.27%)	16 (27.12%)	0.775
Multi-focal	141 (59%)	26 (44.07%)	0.055
Diffuse	40 (16.74%)	17 (28.82%)	0.971
Extent score	4 (2–5)	5 (3–7)	0.057
Density of lesions			
GGO	77 (32.22%)	11(18.64%)	
Mixed (Mainly GGO)	98 (41.00%)	27 (45.76%)	0.606
Mixed (Mainly Consolidation)	57 (23.85%)	20 (33.9%)	0.158
Consolidation	7 (2.93%)	1(1.69%)	0.940
Shape of lesions			
Nodular	1 (0.42%)	1 (1.69%)	0.853
Linear	5 (2.09%)	3 (5.08%)	0.410
Patchy	161 (67.6%)	41 (69.49%)	0.875
Large patchy	72 (30.13%)	14 (23.73%)	
Halo sign	67 (28.03%)	22 (37.29%)	0.218
Reverse halo sign	11 (4.60%)	2 (3.39%)	0.958
Reticulation	61 (25.52%)	11 (18.64%)	0.349
Air bronchogram	85 (35.56%)	26 (44.07%)	0.289
Bronchiectasis	25 (10.46%)	2 (3.39%)	0.150
Vascular enlargement	82 (34.31%)	21 (35.59%)	0.974
Pleural thickening	101 (42.26%)	27 (45.76%)	0.734
Pleural traction	60 (25.10%)	15 (25.42%)	1.000
Pleural effusion	12 (5.02%)	6 (10.17%)	0.237
Mediastinal Lymphadenopathy	23 (9.62%)	7 (11.86%)	0.787
Liver-spleen CT value ratio	1.17 (1.05–1.27)	1.19 (1.07–1.37)	0.278

GGO: Ground-glass opacities

**Table 3:** Univariate logistic regression analysis of features for differentiating COVID-19 patients and non-COVID patients in Primary cohort

Features	Non-COVID-19 (n = 82)		COVID-19 (n = 157)		Coefficient		OR	*P* value
Clinical characteristics								
Age, mean ± SD	49.29 ± 17.49		44.75 ± 14.82		− 0.02		0.98	0.037*
Gender, male/female	50/32		84/73		− 0.31		0.74	0.270
Epidemiological history^#^								
Direct contact	1 (1.22%)		68 (43.31%)		4.13		61.89	< 0.001*
Indirect contact	3 (3.66%)		39 (24.84%)		2.16		8.70	< 0.001*
None contact	78 (95.12%)		50 (31.85%)		− 3.73		0.02	< 0.001*
Symptom								
Fever	42 (51.22%)		126 (80.25%)		1.35		3.87	< 0.001*
Cough	24 (29.27%)		82 (52.23%)		0.47		1.60	0.084
Chest distress	9 (10.98%)		18 (11.46%)		0.05		1.05	0.910
Diarrhea	1 (1.22%)		4 (2.55%)		0.75		2.12	0.505
Anorexia	1 (1.22%)		5 (2.55%)		0.75		2.12	0.505
Laboratory Test, mean ± SD								
WBC, × 10^9^/L		8.72 ± 4.15		5.068 ± 1.80		− 0.54	0.58	< 0.001*
Lymphocyte count, × 10^9^/L		1.42 ± 0.68		1.18 ± 0.47		− 0.77	0.46	0.002*
LDH, U/L		231.78 ± 109.50		250.66 ± 72.02		0.003	1.00	0.114
CRP, mg/L		31.08 ± 40.56		23.06 ± 29.40		− 0.01	0.99	0.089
PCT, ng/mL		0.91 ± 4.28		0.07 ± 0.13		− 3.56	0.03	0.002*
ALT, U/L		47.80 ± 32.60		38.51 ± 61.19		− 0.003	1.00	0.226
AST, U/L		44.95 ± 40.05		34.38 ± 43.01		− 0.01	0.99	0.091
Imaging manifestation								
Involved lobes								
Right Upper Lobe		35 (42.68%)		109 (69.43%)		1.12	3.05	< 0.001*
Right Middle Lobe		36 (43.90%)		93 (59.24%)		0.62	1.86	0.025*
Right Lower Lobe		48 (58.54%)		131 (83.44%)		1.27	3.57	< 0.001*
Left Upper Lobe		36 (43.90%)		107 (68.15%)		1.01	2.73	0.001*
Left Lower Lobe		42 (52.44%)		123 (78.34%)		1.62	5.03	< 0.001*
Main distribution								
Anterior Part of Lungs		19 (23.17%)		25 (15.92%)		− 0.47	0.63	0.172
Posterior Part of Lungs		45 (54.88%)		123 (78.34%)		1.06	2.88	< 0.001*
Location of lesions^#^								
Peripheral		49 (59.76%)		109 (69.43%)		0.43	1.53	0.135
Central		12 (14.63%)		4 (2.55%)		− 1.88	0.15	0.002*
Both		21 (25.61%)		44 (28.02%)		0.12	1.13	0.690
Extent of lesions^#^								
Unifocal		41 (50.00%)		17 (10.83%)		− 2.11	0.12	< 0.001*
Multi-focal		28 (34.15%)		113 (71.97%)		1.60	4.95	< 0.001*
Diffuse		13 (15.85%)		27 (17.20%)		0.10	1.10	0.792
Extent score		4.41 ± 5.32		5.48 ± 3.59		0.07	1.07	0.072
Density of lesions^#^								
GGO		35 (42.68%)		42 (26.75%)		− 0.71	0.49	0.013*
Mixed (Mainly GGO)		26 (31.70%)		72 (45.86(%)		0.60	1.82	0.036*
Mixed (Mainly Consolidation)		18 (21.95%)		39 (24.84%)		0.16	1.18	0.619
Consolidation		3 (3.66%)		4 (2.54%)		− 0.37	0.69	0.631
Shape of lesions^#^								
Nodular		0 (0%)		1 (0.63%)		13.92	1,113,402.31	0.987
Linear		0 (0%)		5 (3.18%)		14.95	3,106,188.55	0.982
Patchy		56 (68.29%)		106 (66.88%)		− 0.07	0.94	0.825
Large patchy		26 (31.71%)		46 (29.30%)		− 0.11	0.89	0.700
Halo sign		22 (26.83%)		45 (28.66%)		0.09	1.10	0.765
Reverse halo sign		2 (2.44%)		9 (5.73%)		0.89	2.43	0.263
Reticulation		11 (13.41%)		50 (31.85%)		1.10	3.02	0.040*
Air bronchogram		22 (26.83%)		63 (31.85%)		0.60	1.83	0.043*
Bronchiectasis		8 (9.76%)		17 (10.83%)		0.12	1.12	0.797
Vascular enlargement		14 (17.07%)		68 (43.31%)		1.31	3.71	< 0.001*
Pleural thickening		17 (20.73%)		84 (53.50%)		1.48	4.40	< 0.001*
Pleural traction		16 (19.51%)		44 (28.03%)		0.47	1.61	0.152
Pleural effusion		9 (10.98%)		3 (1.91%)		− 1.85	0.16	0.007*
Mediastinal Lymphadenopathy		20 (24.39%)		3 (1.91%)		− 2.81	0.06	< 0.001*
Liver-spleen CT value ratio		1.18(1.02–1.29)		1.17 (1.06–1.35)		0.11	1.12	0.826

^*^P value < 0.05 indicates statistical significance

^#^Set as dummy variables in feature selection and Logistic model analysis

WBC: White blood cell count; LDH: Lactate dehydrogenase; CRP: C-reactive protein; PCT: Procalcitonin; ALT: Alanine aminotransferase; AST: Aspartate aminotransferase

### Laboratory tests

Compared with the non-COVID-19 group, COVID-19 group showed lower WBC (*P* < 0.001) and lymphocyte count(*P* = 0.002), as well as lower levels of PCT(*P* = 0.002) (Table 3).

### Chest CT imaging findings

Imaging characteristics were assessed and compared between two groups (Tables 3). Regarding the location and the distribution of the lesions, COVID-19 patients were found to be more located in posterior part of the lungs (*P* < 0.001) compared with non-COVID-19 patients. They had more involvement in every lobe of the lung (*P* < 0.05) due to more multifocal distribution (*P* < 0.001). Besides, they were more likely to have specific signs including reticular changes (*P* = 0.04), vascular enlargement (*P* < 0.001), air bronchogram (*P* = 0.043), and pleural thickening (*P* < 0.001). They were less likely to show pleural effusion (OR 0.16, *P* = 0.007) or mediastinal lymphadenopathy (*P* < 0.001). Other parameters were not significantly different.

### Feature selection

**Fig. 2:**
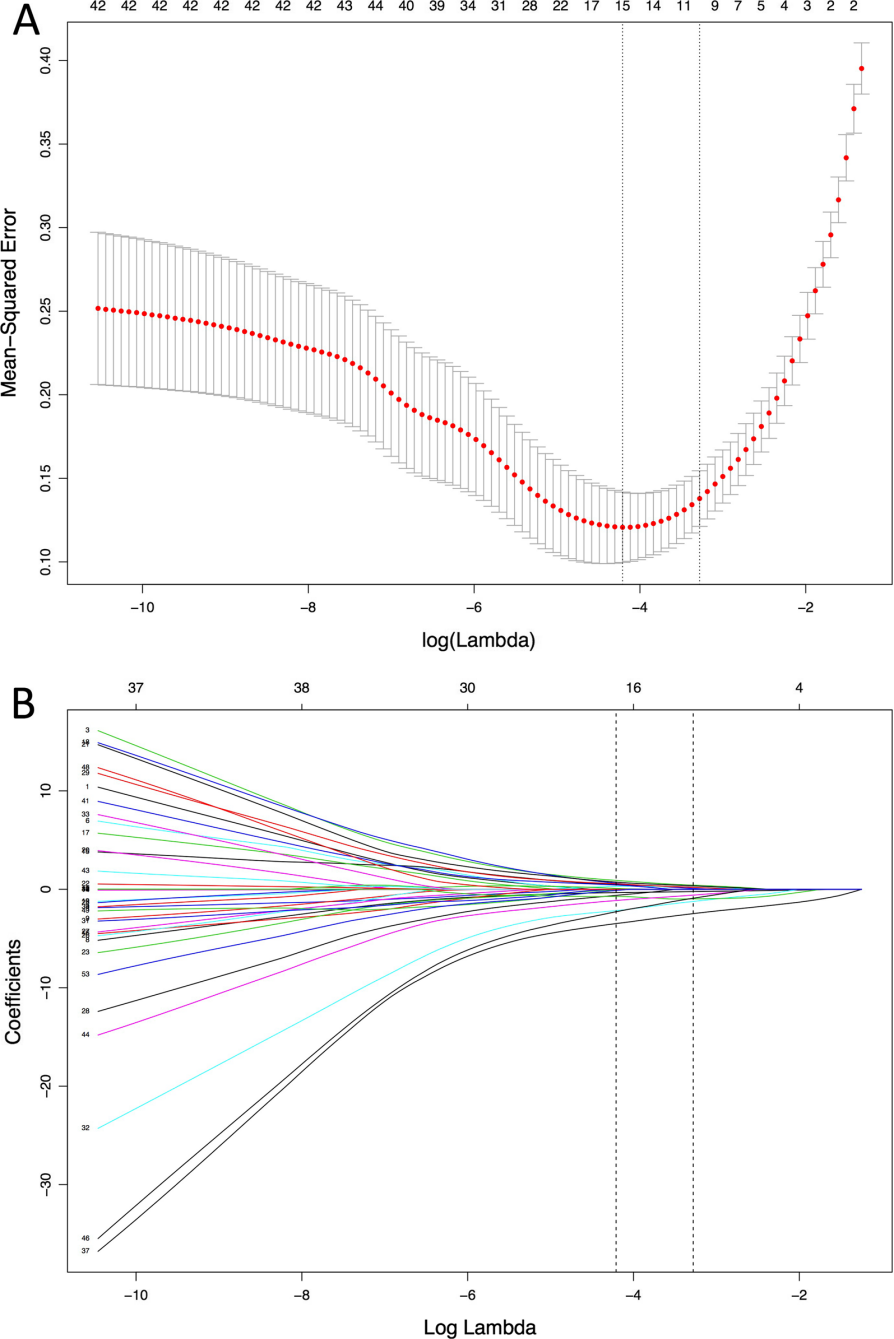
Feature selection using the least absolute shrinkage and selection operator (LASSO) binary logistic regression model. **A** The parameter (λ) in the LASSO model used tenfold cross-validation based on minimum criteria. The mean squared error was plotted versus log(λ). Dotted vertical lines were drawn at the optimal values by using the minimum criteria and the 1 standard error of the minimum criteria (the 1-SE criteria). **B** The plot of LASSO coefficient profiles was produced against the log (λ) sequence. The dotted vertical line was drawn at the optimal values by using the minimum criteria and the 1 standard error of the minimum criteria (the 1-SE criteria), and the latter was chosen with the λ value of 0.0376 and log (λ) of − 3.280 according to the tenfold cross-validation that resulted in 11 nonzero coefficients

In LASSO model, the λ value of 0.0376 with log (λ) of -3.280 chosen (1-SE criteria), and a total of 38 features were reduced to 11 potential features with nonzero coefficients on the basis of 239 patients (21.7:1 ratio; Fig. 2). These features were further incorporated in the multivariate logistic analysis (Table 4). Eight features were found to be statistically significant. COVID-19 group tended to have more fever (OR 4.22; 95% CI [confidence interval], 1.09–18.63; *P* = 0.043), less probability of no contact history (meaning higher probability of indirect or direct contact history [OR 284.73; 95% CI, 38.17–4214.18; *P* < 0.001]), lower WBC count (OR 0.63; 95% CI, 0.47–0.77; *P* < 0.001), more involving left lower lobe (OR 9.42; 95% CI, 1.95–62.80; *P* = 0.010), more exhibiting multifocal lesions (OR 8.98; 95%CI, 1.58–61.36; *P* = 0.017), more pleural thickening (OR 5.59; 95%CI, 1.32–28.85; *P* = 0.026), less located in central part (OR 0.09; 95%CI, 0.01–0.75; *P* = 0.043), and less mediastinal lymphadenopathy (OR 0.037; 95% CI, 0.00–0.29; *P* = 0.004).

**Table 4:** Multivariate logistic regression analysis of features for differentiating COVID-19 patients and non-COVID patients

Features	Coefficient	OR	95%CI	*P* value
Fever	1.44	4.22	(1.09,18.63)	0.043*
Epidemiological history: None contact	− 5.65	0.00	(0.00,0.03)	< 0.001*
WBC count	− 0.47	0.63	(0.48,0.77)	< 0.001*
Lesion involvement: Unifocal	0.11	1.12	(0.12,10.58)	0.919
Lesion involvement: Multi-focal	2.19	8.98	(1.59,61.36)	0.017*
Involved lobes: Right Upper lobe	1.12	3.05	(0.75,13.21)	0.121
Involved lobes: Left Upper Lobe	0.77	2.16	(0.51,9.52)	0.295
Involved lobes: Left Lower Lobe	2.24	9.42	(1.95,62.80)	0.010*
Pleural thickening	1.72	5.59	(1.32,28.85)	0.026*
Mediastinal lymphadenopathy	− 3.30	0.04	(0.00,0.29)	0.004*
Distribution Central	− 2.45	0.09	(0.01,0.75)	0.043*

^*^P value < 0.05 indicates statistical significance

Abbreviations: WBC: White blood cell count

### Nomogram

**Fig. 3:**
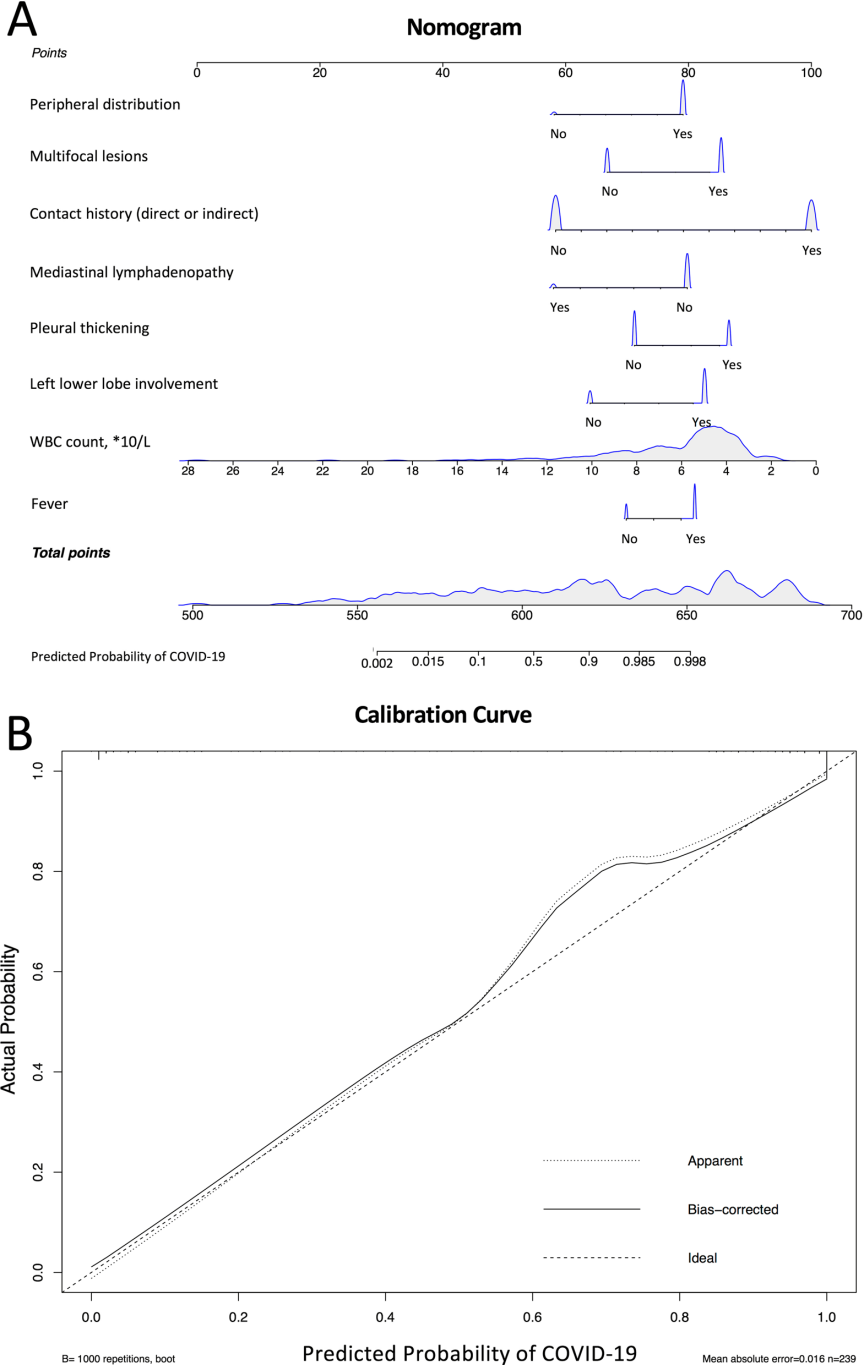
The nomogram and calibration curves based on significant features in multivariate analysis. **A** A nomogram was built on the basis of eight significant features in multivariate Logistic model. If a patient is suspected to be COVID-19 by radiological diagnosis, the data needed includes whether he has fever, contact history, decreased WBC count, left lower lobe involvement, pleural thickening, multifocal lesions, peripheral distribution or absence of mediastinal lymphadenopathy. The point of each feature adds up to a total score with a corresponding probability of COVID-19. **B** The calibration curve was determined with bootstrap analysis to get bias-corrected estimation. It indicated great agreement between the prediction and the actual grouping in the probability

**Fig. 4:**
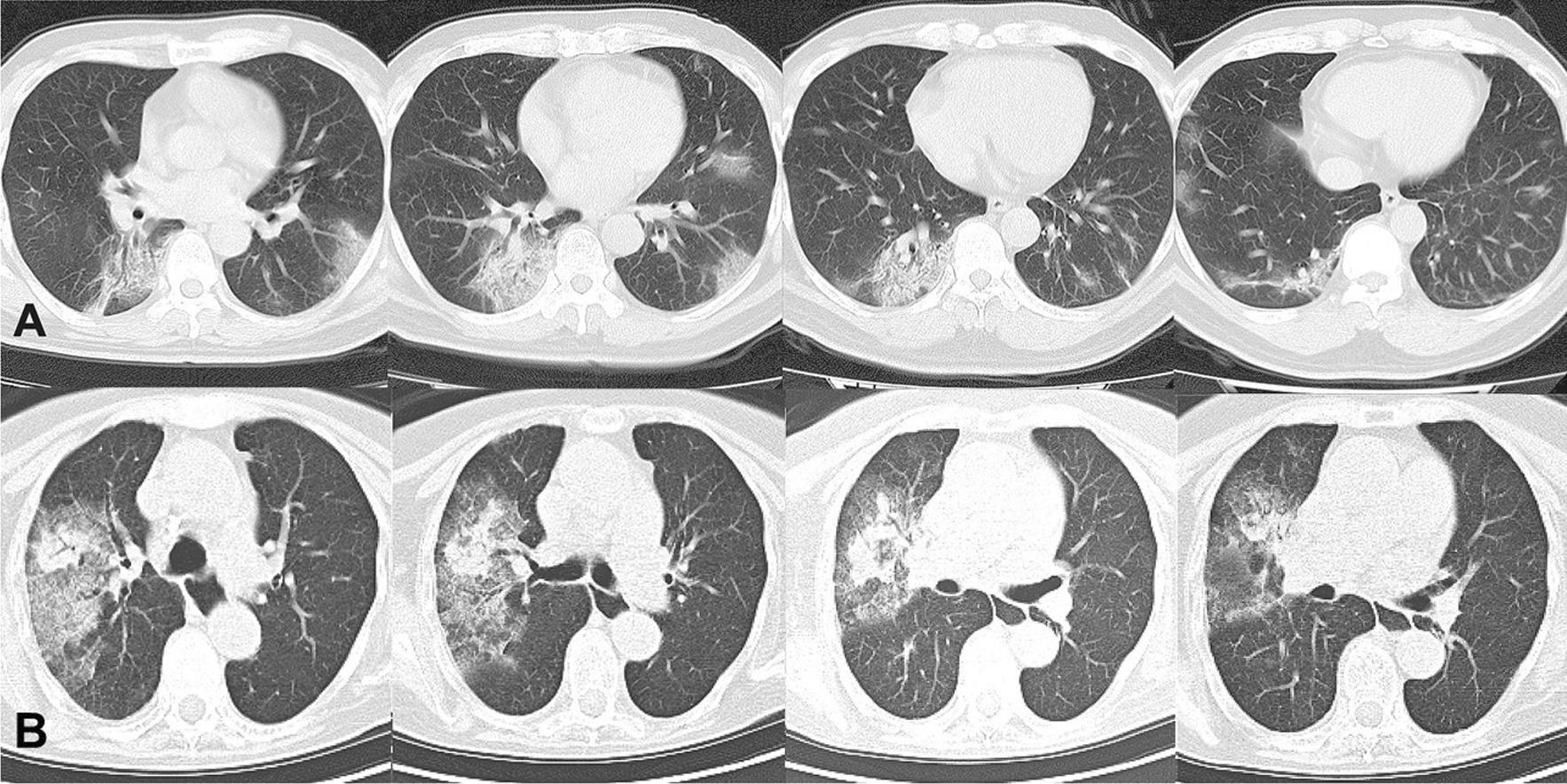
Two representative cases to illustrate the application of the nomogram. **A** A 40-year-old male patient complained of fever for 4 days (score ≈ 80). He had travelled to Huangshi, a city in Wuhan Province, China a week before the onset (score ≈ 100). His laboratory tests indicated leukocytopenia (1.99*10^9/L, score ≈ 92). His chest CT showed patchy ground glass opacities with vascular enlargement and reticular changes on bilateral lower lobes (left lower lobe involvement: score ≈ 83; multifocal: score ≈85). Lesions were located both central and peripheral (score ≈ 80). No mediastinal lymphadenopathy was observed (score ≈ 80). Slight pleural thickening was observed (score ≈ 85). Total estimated score reached around 687, indicating > 99.8% probability to be a COVID-19 case. He was later confirmed by RT-PCR. **B** A 60-year-old female patient complained of fever for 3 days (score ≈ 80). She claimed no contact or exposure history (score ≈ 60). Her WBC count is slightly elevated (10.52*10^9/L, score ≈ 60). Her chest CT showed unifocal (score ≈ 68) large patchy ground glass opacities with consolidation only involving the right upper lobe (score ≈ 63), but with both central and peripheral distribution (score ≈ 80). Mediastinal lymphadenopathy was observed in mediastinal window (score ≈ 60). No pleural thickening (score ≈ 70). Total estimated score reached around 541, indicating < 0.2% probability to be a COVID-19 case. She was radiologically suspected as COVID-19, but the diagnosis of COVID-19 was ruled out by 2 consecutively negative RT-PCR test results. She was finally diagnosed with respiratory syncytial virus infection

A nomogram was constructed based on the multivariate Logistic analysis model. The adjusted C-index of the nomogram was 0.97 (Fig. 3A). The calibration curve was determined with bootstrap analysis to get bias-corrected estimation. It indicated great agreement between the prediction and the actual diagnosis in the probability (Fig. 3B). The HL goodness-of-fit test showed good calibration as well (*P* = 0.4797). The CT images of two cases illustrated the application of the nomogram (Fig. 4).

### External validation

The validation cohort included 59 cases with 43 COVID-19 and 16 non-COVID. The baseline data were collected in Tables 1 and 2. 56 out of 59 cases were correctly predicted using the nomogram, reaching an accuracy of 94.91%. Calibration was good (*P* = 0.9956 for the HL goodness-of-fit test).

## Discussion

An ongoing outbreak of COVID-19 originated from Hubei Province in China has been spreading worldwide. Experts in infectious and respiratory diseases, critical care, and radiology from all over the world have been making a joint effort to contain the epidemic situation [18]. Presently, RT-PCR is the standard confirmative method in spite of a few flaws including long turnaround time for the results in underdeveloped regions and low sensitivity especially in the early phase of the disease [10]^1920^. On the contrary, chest CT scan is able to recognize the lesions at earlier stages with high sensitivity, thus is considered an important tool to guarantee an early diagnosis and isolation of infected patients [8]. Before the RT-PCR results are attainable, the quarantine is needed, but the isolation site is insufficient, and it possibly delays essential treatment. In this study, the CT manifestations summarized by the National Health Commission of China were used as the inclusion criteria. We investigated the differential values of clinical characteristics, laboratory results and CT features to better distinguish COVID-19 patients from those with suspicious CT findings, and developed a model with a nomogram as a practical tool.

The most common symptom in the patients we enrolled is fever, followed by cough and chest distress. As a differential feature, fever is significant in both univariate and multivariate analysis. This echoes previous studies, and fever is the leading symptom listed in the case definition for surveillance of COVID-19 by the Chinese Health Commission [17, 21, 22]. Therefore, it is necessary to monitor body temperature and at-home temperature measurement is a useful and easy way for the public to early notice. Additionally, we noticed a small portion of the patients with digestive disorders like diarrhea and anorexia, and it occurred more in the COVID-19 group. Increasing evidence shows the manifestation of COVID-19 is not always confined to respiratory symptoms, but may also involve other systems, e.g., the central nervous system [23, 24]. Liver function abnormalities have been reported in COVID-19 patients with a pooled prevalence of 19% (95% confidence interval, 9–32%) with an association with disease severity. Hepatocyte degeneration, focal necrosis, and fatty infiltration were reported in COVID-19 patients [25, 26]. LS ratio was observed in this study since the CT values were attainable in chest CT image, but was insignificant here.

The contact history is another valuable factor for COVID-19, including direct contact with COVID-19 patients, direct exposure in districts with confirmed cases, and indirect contact with those who were exposed [27]. According to the National Health Commission of China, a patient with one exposure or contact history and two clinical conditions can be regarded as a suspected case [17]. However, with the swift spread of the disease, some contact history is unrevealed, making it harder to contain the epidemic [28]. More active precaution and isolation is needed.

Among the laboratory parameters, WBC count is significantly lower in COVID-19 group in both univariate and multivariate analysis, and lymphocyte count is lower in univariate analysis. This is consistent with previous findings and the criteria by the ﻿Chinese Health Commission [1, 12, 17]. We also found lower levels of CRP and PCT in the COVID-19 group. They are useful indicators of infection or inflammation, and CRP was previously reported to increase in COVID-19 patients by some researchers [8, 29]. Our finding may result from higher extent of increased levels of these indices in non-COVID-19 patients since they had other inflammatory conditions including bacterial infection, while other studies used healthy controls. Typical radiographic features on chest CT in COVID-19 patients were reported to predominantly include bilateral and peripheral GGOs and consolidative pulmonary opacities. The location of the lesions varied among studies, yet the peripheral site is most frequently reported [8, 30–32]. These widely-accepted imaging characteristics constituted the most important inclusion criterium in this study, thus were seen in both groups. Less typical signs in previous studies included linear opacities, "crazy-paving" pattern and the reverse halo sign, etc. [8, 33–37]. We found that COVID-19 lesions are more commonly seen in both lower lobes, which echoes existing literature. We also found that the right lower lobe was more often involved in both COVID-19 and non- COVID-19 groups, which may be related to the shorter and thicker structure of the right lower lobe bronchus that may make it easier for the pathogens to enter this lobe [38]. There are also studies that found left lower lobe to be mostly involved [39, 40]. Distribution in all lobes showed significant difference between two groups, but left lower lobe involvement remained after two-step feature selection, making it a significant feature in differentiating COVID-19 patients from other conditions. Although it is unclear at this time why it is useful, further investigations of the common distribution and the corresponding mechanisms of the diseases in the non-COVID-19 group respectively will be helpful. Besides, compared with non-COVID-19 cases, COVID-19 is more likely to exhibit multifocal distribution rather than unifocal changes, and more likely to have reticulated changes, vascular enlargement, and pleural thickening. The pooled prevalences of pleural thickening in COVID-19 patients were 30.0–52.46% [39, 41, 42]. COVID-19 patients are also less likely to have pleural effusion and mediastinal lymphadenopathy, which is consistent with prior researches [30].

Fever, contact history, decreased WBC count, left lower lobe location, pleural thickening, multifocal lesions, peripheral distribution, and absence of mediastinal lymphadenopathy were found to be features independently associated to COVID-19 patients. On the basis of these parameters, a nomogram was built to better interpret our findings, which is popular in cancer research these years [35]. According to our nomogram, the point of each feature adds up to a total score with a corresponding probability of COVID-19.

A nomogram can be validated by both internal and external validation [36]. In this study, internal validation used the data of the same cohort for the generation of the nomogram, and external validation used the data from another institution. Both internal and external validation indicated good agreement between the prediction and the actual diagnosis in the probability.

Since the COVID-19 outbreak, the scientific researchers have focused more on clinical and radiological findings of COVID-19 infection, whereas a few studies have investigated the differential diagnoses. Three studies from Europe presented a vast spectrum of differential diagnoses with abundant figures and elaborate illustrations to help the radiologist with differentiation [43–45]. Another study evaluated the performances of radiologists from US and China in differentiating COVID-19 from other viral pneumonia [46]. Researchers from Japan compared COVID-19 and other diseases with similar symptoms, and proposed useful laboratory indicators [47]. The studies above investigated the differential diagnosis of COVID-19, but did not construct a practical model. One study built a diagnostic model, but with a small sample size, and only included non-COVID-19 pneumonia patients in the control group [48]. Our study has a different design from those of existing papers. In this study, the typical CT manifestations of COVID-19 were used as the inclusion criteria, thus a wider spectrum of diseases that needed to be differentiated from COVID-19 was included, which is a realistic problem that may be encountered in clinical practice.

In summary, this study is the first to investigate the features to distinguish confirmed COVID-19 patients from other conditions with similar CT findings, which is an important clinical issue. The nomogram can be used as an instant tool able to provide practical reference for individualized management for every suspected patient and is likely to offer effective and scientific basis for empirical treatment.

Our study had several limitations. Firstly, in this multi-center study, the normal range and results of the laboratory data might be different due to the differences in the kits, equipment, and environmental conditions. However, three institutions are all China's Grade-A Tertiary Hospitals, with laboratories of the highest qualifications, and similar protocols are adhered, thus the results are relatively stable. Secondly, the sample size is relatively small since no data was obtained from the epicenter of the outbreak, and the spread of COVID-19 was successfully suppressed in a few months in China as appropriate precautions were taken. Besides, despite being the standard confirmative test, RT-PCR has false-negative probabilities, therefore our results might be biased since non-COVID-19 group might include infected patients. Future prospective investigation of larger scale with international data and evolved diagnostic techniques is expected.

## Conclusion

In conclusion, fever, contact history, decreased WBC count, left lower lobe involvement, pleural thickening, multifocal lesions, peripheral distribution, and absence of mediastinal lymphadenopathy are able to distinguish COVID-19 patients from other suspected patients. The nomogram based on these features is a useful tool in the clinical practice.

## Data Availability

The datasets generated and/or analysed during the current study are not publicly available due to ethical restrictions but are available from the corresponding author on reasonable request.

## Abbreviations

COVID-19: Coronavirus disease 2019
SARS-CoV-2: Severe Acute Respiratory Syndrome Coronavirus 2
WHO: World Health Organization
PHEIC: Public Health Emergency of International Concern
NAAT: Nucleic acid amplification test
MIS-C: Multiple system inflammatory syndrome
RT-PCR: Reverse transcriptase-polymerase chain reaction
CT: Computed tomography
IRB: Institutional Review Board
WBT: White blood count
CPR: C-reactive protein
LDH: Lactate dehydrogenase
PCT: Procalcitonin
GGO: Ground-glass opacity
RHS: Reversed halo sign
ROI: Region of Interest
LASSO: Least absolute shrinkage and selection operator
OR: Odds ratio
LR ratio: Liver/spleen ratio
95% CI: 95% Confidence interval
